# Comparison of the effects of salmeterol/fluticasone propionate with fluticasone propionate on airway physiology in adults with mild persistent asthma

**DOI:** 10.1186/1465-9921-8-52

**Published:** 2007-07-14

**Authors:** Catherine M Houghton, Naomi Lawson, Zoe L Borrill, Claire L Wixon, Sally Yoxall, Stephen J Langley, Ashley Woodcock, Dave Singh

**Affiliations:** 1 North West Lung Research Centre, South Manchester University Hospitals Trust, Manchester, UK; 2 Research and Development, GlaxoSmithKline, Greenford, Middlesex, UK; 3 Faculty of Medical and Human Sciences, The University of Manchester, Manchester, UK

## Abstract

**Background:**

This study compared the effect of inhaled fluticasone propionate (FP) with the combination of salmeterol/fluticasone propionate (SFC) on lung function parameters in patients with mild asthma.

**Methods:**

Adult patients with mild persistent asthma (≥ 80% predicted FEV_1_) receiving 200–500 μg of BDP or equivalent were randomised to receive either FP 100 μg or SFC 50/100 μg twice daily from a Diskus^® ^inhaler for four weeks. The primary outcome was the change from baseline in airway resistance (sRaw) at 12 hrs post dose measured by whole body plethysmography. Impulse oscillometry and spirometry were also performed.

**Results:**

A comparison of the geometric mean sRaw at 12 hrs post dose in the SFC group to the FP group gave a ratio of 0.76 (0.66 – 0.89, p < 0.001) at week 2 and 0.81 (0.71 – 0.94, p = 0.006) at week 4. Similarly, significant results in favour of SFC for oscillometry measurements of resistance and reactance were observed. FEV_1 _was also significantly superior at week 2 in the SFC group (mean difference 0.16L, 95% CI; 0.03 – 0.28, p = 0.015), but not at week 4 (mean difference 0.17L, 95% CI -0.01 – 0.34, p = 0.060).

**Conclusion:**

SFC is superior to FP in reducing airway resistance in mild asthmatics with near normal FEV_1 _values. This study provides evidence that changes in pulmonary function in patients with mild asthma are detected more sensitively by plethysmography compared to spirometry

**Trial registration number:**

NCT00370591.

## Background

Clinical trials of drug treatments for asthma commonly use the spirometric assessment of FEV_1 _to assess improvements in lung function. FEV_1 _is a relatively simple and reproducible measurement that is required by regulatory authorities. However, in patients with mild asthma, FEV_1 _may be close to normal and is not as sensitive as body plethysmography and impulse oscillometry (IOS) for measuring small changes in lung function in response to broncodilators [1-3]. Furthermore, body plethysmography assesses airway resistance while IOS measures pulmonary resistance and reactance, properties not assessed by spirometry [3-6].

Combination therapies of inhaled corticosteroids (ICS) with a long acting beta agonist (LABA) are effective in the treatment of asthma. The combination of salmeterol and fluticasone propionate (SFC, Seretide GSK™) improves symptoms, lung function and exacerbation rates when compared to the same or double the dose of fluticasone propionate (FP) in symptomatic patients with asthma who have moderately impaired lung function (FEV_1 _<80% predicted) [7-9]. However, there are less data regarding the effects of SFC in symptomatic patients with mild asthma who may have almost normal FEV_1 _values. The advantages of SFC over ICS alone in this subgroup is difficult to evaluate using FEV_1_, as this measurement is near normal. In the absence of studies using more sensitive pulmonary function measurements than FEV_1_, guidelines have favoured the use of ICS alone for mild asthma patients [10]. However, the benefits of combination therapy with SFC in patients with mild asthma may be more apparent if sensitive pulmonary function measurements, such as body plethysmography and IOS are used.

We report a comparison of the effects of SFC 50/100 μg twice daily compared to FP 100 μg bd in patients with mild asthma already being treated with ICS. Our primary aim was to compare the changes in lung function, assessed by plethysmography, IOS and spirometry.

## Methods

### Study subjects

Patients with physician diagnosed asthma for at least six months who were receiving a stable total daily dose of ICS equivalent to 200–500 μg beclomethasone dipropionate (BDP) for at least 4 weeks prior to the study were enrolled. The inclusion criteria included an FEV_1_≥ 80% predicted and demonstration of a ≥ 30% decrease in sRaw in response to 400 μg of inhaled salbutamol at screening. This reversibility criterion was based on previous data demonstrating that this magnitude of change in sRaw was significantly greater than within day variability [1]. Exclusion criteria were the use of parenteral, oral and nebulised steroids in the 4 weeks prior to the study or 12 weeks for depot corticosteroids and current smokers or ex smokers for < 12 months. All female patients of childbearing potential were required to be using appropriate contraception and have a negative pregnancy test results at screening. All patients gave written informed consent and the study was approved by the south manchester ethics committee; reference number 02/SM/460.

### Study design and medication

**Figure 1:**
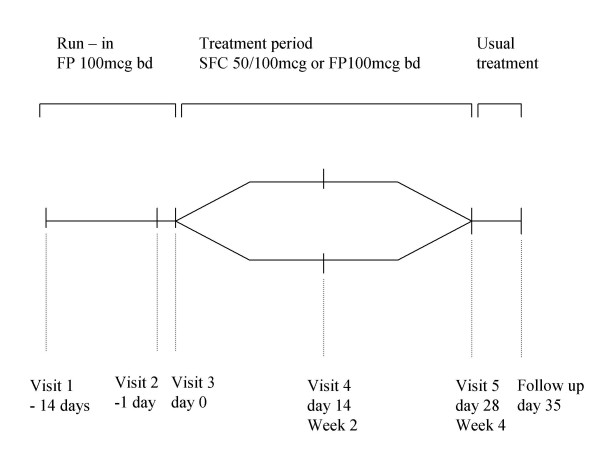
Study flow chart.

This was a single centre, randomised, double blind, parallel group trial conducted between December 2002 and April 2004. All patients were treated with FP 100 μg bd through an Accuhaler/Diskus (GSK™) during a 2 week run in period. To be eligible for randomisation, subjects were required to have symptoms more than once a week, but not every day of the week during the run in period. At the end of the run in period, baseline measurements of pulmonary function and methacholine reactivity were performed. Subjects were then randomised to SFC 50/100 μg bd or FP 100 μg bd via identical Accuhaler devices for a 4 week treatment period (Figure 1). Patients were provided with a salbutamol Accuhaler for as required use. Pulmonary function measurements were performed after the run in period (baseline) and after 2 and 4 weeks of treatment. These were performed in the morning before dosing with study medication (*pre-dose*), and at 2 hrs *post-dose*. No IOS data was collected at week 2. Methacholine reactivity was assessed 2 hrs post-dose at week 4. Patients were given diary cards in which to record morning and evening peak expiratory flow (PEF).

### Pulmonary function and methacholine challenge tests

Pulmonary function tests were always performed in the same order: (1) IOS, (2) plethysmography and (3) spirometry. The deep inspiration required for spirometry may cause a temporary alteration in bronchial tone [11] so resistance measures were made prior to spirometry. A full explanation and training in the performance of each lung function test was given to each subject prior to the study.

For IOS (Masterscreen IOS, Erich Jaeger, Hoechberg, Germany) subjects supported their cheeks to reduce upper airway shunting while impulses were applied during tidal breathing for 30 seconds. The mean of three readings of R5 and R20 (respiratory resistance at 5 and 20 Hz respectively), X5 (reactance at 5 Hz) and RF (resonant frequency) were recorded. For plethysmography, sRaw and sGaw were measured in a constant volume plethysmograph (Sensormedics Vmax 6200) and the mean of 3 readings was recorded. For spirometry, FEV_1 _(volume expired over the first second) and MMEF (maximal mid expiratory flow rate) were performed (Masterscreen, Erich Jaeger) and the mean of 3 readings was recorded.

Methacholine challenge tests were performed as previously reported [12]. A De Vilbiss 646 nebulizer (Sunrise Medical; Wollaston, UK) and a Rosenthal dosimeter (PDS Research UK; Gravesend, UK) were used to deliver the methacholine. Three concentrations of methacholine chloride were used (1.5 mg/ml, 12 mg/ml and 50 mg/ml, Stockport Pharmaceuticals, UK) to administer doubling doses, starting from a dose of 0.015 mg increasing to a final cumulative dose of 5.96 mg. The PD_20 _was calculated as thecumulative dose that produced a 20% decrease in FEV_1 _by interpolation.

### Statistical analysis

The primary outcome measure was the morning pre-dose sRaw after 4 weeks treatment with SFC and FP. Based on previous studies a 20% difference in pre dose sRaw between the two treatment groups was identified as a clinically relevant difference [13]. A sample size of 18 evaluable subjects per group (36 evaluable subjects in total) would have 90% power to detect a difference of 20% between treatment groups in pre-study medication sRaw at 4 weeks of treatment, assuming a common standard deviation of 0.20 s.kPa of natural log sRaw, at a 5% two-sided significance level. A sample size of 20 subjects per group would allow for an estimated treatment withdrawal rate of 10%.

The differences between the effects of SFC and FP on pulmonary function measurements and methacholine reactivity were analysed by ANCOVA with covariates of age, gender, baseline measurement and treatment group. sRaw and sGaw were log transformed and least square means for the treatments were transformed back to the original scale and are presented as geometric means. Baseline PEF was defined as the mean of the daily values over the last 7 days of the 2 week run in period. Mean morning and evening PEF were calculated from all the available data from week 1 to 4. Statistical analysis was performed on an Intention To Treat basis with all subjects randomised to treatment being included. If there was no data for the primary endpoint (sRaw) at week 4, the last observation from week 2 was carried forward. For analysis of bronchial hyperreactivity, only subjects who had a 20% decrease in FEV_1 _before or at the highest methacholine concentration at the end of the run in period and at week 4 (i.e. those with PD_20_≤ 5.96 mg) were included.

## Results

Fifty six patients were enrolled into the run in period, of which 13 did not meet the daily symptom score requirement, 3 did not complete the diary card correctly and 1 was non compliant with run in medication. The demography and lung function at screening (before run in) of the remaining 39 subjects who were eligible for randomisation is shown in Table 1. Baseline measurements of pulmonary function after the run in are shown in Table 2. There were 2 withdrawals following randomisation, one in each treatment group due to loss of study medication in he SFC group and medication running out in the FP group.

**Table 1:** Subject demographics at screening

	**SFC 50/100** **N = 19**	**FP 100** **N = 20**
Age	38.4 (9.8)	41.8 (14.7)
Sex ^1^		
Female	10 (53%)	12 (60%)
Male	9 (47%)	8 (40%)
Duration of asthma ≥ 15 years	11 (58%)	7 (35%)
% predicted FEV_1_	93.4 (9.0)	95.3 (11.4)
FEV_1_	3.22 (0.55)	3.04 (0.81)
sRaw s.kPa*	1.00 (39.61)	0.94 (50.28)
sGaw s^-1 ^kPa	1.29 (152.11)	1.07 (50.47)
% change in sRaw post 400 μg salbutamol	-43.6 (10.2)	-38.4 (7.0)

Data is mean (SD) except;

* = geometric mean (CV)

^1 ^= number of subjects (percent)

**Table 2:** Baseline (week 0) pulmonary function

	**SFC 50/100** **N = 19**	**FP 100** **N = 20**
sRaw s.kPa *	0.86 (42.0))	0.91 (42.15)
sGaw s^-1 ^kPa^-1^*	1.17 (42.10)	1.11 (41.91)
FEV_1 _(L)	3.24 (0.57)	3.10 (0.75)
MMEF (L.s^-1^)	2.00 (1.45)	2.44 (1.03)
AM PEFR (L/min)	484.02 (105.38)	467.50 (117.64)
PM PEFR (L/min)	494.05 (101.27)	475.52 (112.54)
R5 (kPa.L^-1^.s)	0.44 (0.10)	0.51 (0.15)
R20 (kPa.L^-1^.s)	0.36 (0.08)	0.39 (0.11)
X5 (kPa.L^-1^.s)	0.14 (0.04)	0.17 (0.1)
RF (Hz)	15.29 (4.84)	16.67 (6.35)
AHR (mg) *	0.41 (348.8)	0.37 (372.7)

Data is mean (SD) except;

* = geometric mean (CV)

### Pre-dose pulmonary function

#### Plethysmography

**Figure 2:**
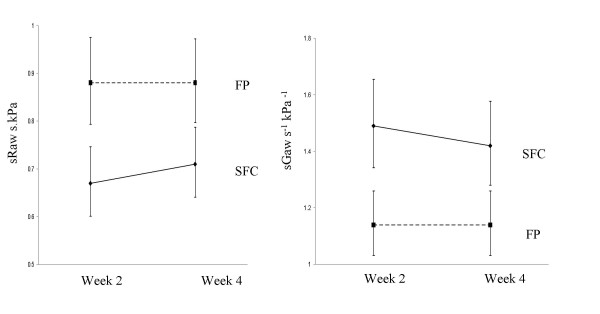
Comparison of sRaw and sGaw between SFC and FP groups. Data points = adjusted geometric mean at week 2 and 4 (ANCOVA adjusted for effects of gender age and baseline lung function). Error bars = 95% confidence intervals

SFC caused a significantly greater reduction in pre dose sRaw compared with FP at weeks 2 and 4 (Figure 2 shows adjusted body plethysmography data). The geometric mean pre dose sRaw was 24% lower in the SFC group compared with the FP group (ratio 0.76, 95% CI; 0.66–0.89, p <0.001) at week 2 and 19% lower (ratio 0.81, 95% CI; 0.71–0.94, p = 0.006) at week 4. Similarly, there was a statistically significant greater increase in pre dose sGaw at week 2 and 4 in the patients receiving SFC compared with those receiving FP (Figure 2). sGaw was 30% greater at week 2 (ratio 1.31, 95% CI; 1.13–1.51, p < 0.001) and 24% greater at week 4 (ratio 1.24, 95% CI: 1.08–1.44, p = 0.004) in the SFC group.

#### Spirometry

**Figure 3:**
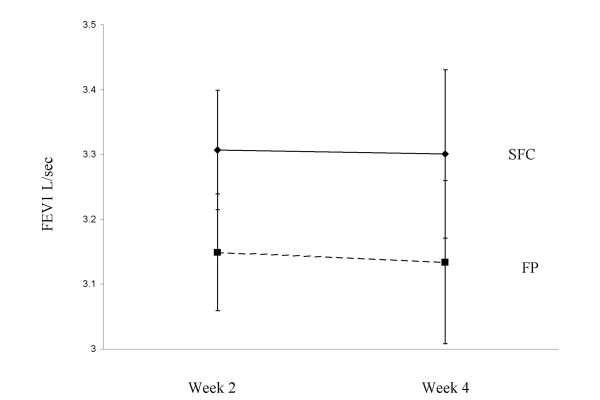
Comparison of FEV_1 _between SFC and FP groups. Data points = adjusted mean change at week 2 and 4 (ANCOVA adjusted for effects of gender age and baseline lung function). Error bars = 95% confidence intervals

At week 2 there was a small increase in the pre-dose FEV_1 _with SFC, that was statistically significant compared with FP; mean difference 0.16L, 95% CI; 0.03–0.28, p = 0.015 (Figure 3 shows adjusted FEV1 data). The mean difference at week 4 was 0.17L, which approached statistical significance (95% CI -0.01–0.34, p = 0.06). There were no significant differences in MMEF measurements between SFC and FP at week 2 (mean difference 1.1L/sec, 95% CI; -0.13–2.32, p = 0.08) and week 4 (mean difference 0.11 L/sec, 95% CI; -0.35 – 0.56, p = 0.6).

#### Impulse Oscillometry

**Figure 4:**
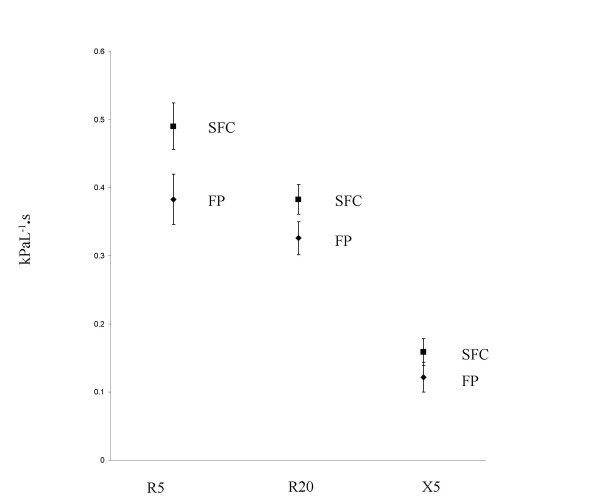
Comparison of R5, R20 and X5 between SFC and FP groups. Data points = adjusted geometric mean at week 4 (ANCOVA adjusted for effects of gender age and baseline lung function). Squares = FP. Diamonds = SFC. Error bars = 95% confidence intervals

At week 4 there were statistically significant improvements in IOS measurements in the SFC group compared with FP (Figure 4 shows adjusted IOS data). The mean RF at week 4 was 13.07 Hz (95% CI; 11.3 – 14.84) in the SFC group and 17.65 (95% CI; 15.99 – 19.3) in the FP group. The mean difference in the resistance parameters R5 and R20 were -0.11 kPaL^-1^.s (95% CI; -0.16 – -0.06, p < 0.001) and -0.06 kPaL^-1^·s (95% CI; -0.09–0.02, p = 0.001) respectively. The mean difference in the reactance parameters RF and X5 were -4.58 Hz (95% CI; -7.01– -2.15, p < 0.001) and -0.04 kPaL^-1^·s (95% CI; -0.07 – -0.01, p = 0.019).

### Post-dose Pulmonary Function

The improvement in lung function at 2 hrs after the first dose was greater in the SFC compared with the FP group; for the primary endpoint of sRaw the adjusted mean changes between pre and post dose were 36.4 % and 7.1 % respectively. The adjusted mean (95% CI) difference was 29.3% (20.7 to 37.9), p < 0.001. Pulmonary function measurements at 2 hrs post dose were stable over the treatment period, with data in both groups after 2 and 4 weeks dosing similar to measurements after the first dose (Table 3).

**Table 3:** Lung function 2 hours post dose

		SFC mean (95% CI)	FP mean (95% CI)
sRaw s.kPa	Week 0	0.53 (0.46, 0.62)	0.84 (0.73, 0.98)
	Week 2	0.56 (0.47, 0.67)	0.85 (0.72, 1.01)
	Week 4	0.56 (0.48, 0.67)	0.77 (0.65, 0.91)
sGaw s^-1 ^kPa ^-1^	Week 0	1.88 (1.61, 2.21)	1.19 (1.02, 1.39)
	Week 2	1.80 (1.51, 2.14)	1.18 (1.00, 1.39)
	Week 4	1.78 (1.50, 2.12)	1.3 (1.10, 1.54)
FEV_1 _(L)	Week 0	3.50 (3.16, 3.84)	3.15 (2.82, 3.48)
	Week 2	3.41 (3.08, 3.73)	3.07 (2.76, 3.38)
	Week 4	3.36 (3.02, 3.70)	3.05 (2.72, 3.38)

### PEF

The mean morning and evening PEF analysed over weeks 1 to 4 was significantly greater (21.6 L/min 95% CI; 10.6 – 32.7, p < 0.001 and 17.6 L/min, 95% CI; 5.1 – 30.2, p = 0.007) in the SFC group compared with the FP group.

### Airway hyperreactivity

Twelve subjects in the SFC group and 16 in the FP group had PD_20 _≤ 5.96 mg at the end of the run in period and at week 4. In these subjects, the ratio (SFC/FP) of the adjusted means for PD_20 _at week 4 was 1.57 (95% CI: 0.70, 3.54), with no difference between groups (p = 0.25).

## Discussion

We have studied patients with mild persistent asthma who are symptomatic despite being treated with ICS. Asthma guidelines advocate a step wise approach to pharmacological therapy in such patients, with the aim of optimizing asthma control defined by a range of clinical endpoints, including symptoms, exacerbation rates and pulmonary function. One possible step wise approach is the use of combined LABA and ICS in these patients. This is the first study to investigate the benefits on airway patency of combination therapy with SFC (50/100 μg bd) compared with ICS (FP 100 μg bd) alone in a group of mild asthma patients.

Our primary endpoint was the sensitive measurement of sRaw using body plethysmography. The key findings were that SFC caused significant improvements in sRaw, sGaw and IOS resistance and reactance parameters at 12 hours post dose after 2 and 4 weeks treatment. At 2 weeks, a small but statistically significant difference was observed using FEV_1_, but not at 4 weeks. In this population of mild asthma patients, selected for reversibility using criteria based on sRaw, we have therefore shown that SFC 50/100 μg has greater benefits on pulmonary function compared with FP 100 μg, which are most apparent when using more sensitive methods than spirometry. Further studies are needed to determine if these pulmonary function findings are true in other asthma populations, and whether overall asthma control is improved by such a strategy.

In order to optimise asthma control in symptomatic mild asthma patients being treated with low dose ICS, it is possible to increase the ICS dose or add in a LABA. Using the SFC combination, we have proved that the addition of LABA has pulmonary function benefits compared to the same dose of ICS alone in this particular population. It would now be important to compare SFC to an increased dose of ICS alone in this population, as these are the 2 treatment options available in clinical practice.

The bronchodilator profile of salmeterol in asthma is well established, with maximal effects observed within 1–2 hrs [13,14]. In this study using SFC, it was therefore relevant to study both the maximal effects (at 2 hrs post dose) as well as the effects at 12 hrs post dose, immediately prior to the next scheduled dose. We chose 12 hrs for the primary endpoint (sRaw) measurement, as this is the time point when the effects of SFC are most likely to be similar to FP. Thus, any superiority of SFC over FP at 12 hrs would indicate that the benefits of SFC are sustained over the full 12 hrs, and not just confined to the first 2 hrs post dose.

Although FEV_1 _is the "gold standard" of clinical trials, we observed that substantial improvements in airway resistance occurred in mild asthmatics without a statistically significant change in FEV_1 _after 4 weeks treatment. We have previously shown that body plethysmography is a sensitive measurement of bronchodilation in mild asthmatics i.e. although body plethysmography has increased variability compared to FEV_1_, it is also more sensitive to changes in airway tone, and so was able to detect changes caused by 10 μg salbutamol while there was no change in FEV_1_[1]. The current study provides further evidence that changes in pulmonary function in patients with mild asthma are detected more sensitively by plethysmography compared to spirometry [1,2].

We did not measure FEV1 reversibility at screening. In the SFC group, there was an improvement in pulmonary function of 260 mls after the first dose, equivalent to 8% reversibility. A higher degree of FEV1 reversibility would not be expected, as the baseline FEV1 was approximately 94% predicted.

Body plethysmography is more time consuming compared to spirometry, and is a more complex technique that requires a greater degree of operator training. However, it is clear that body plethysmography offers advantages in terms of sensitivity and we encourage its use in clinical trials of mild asthmatics [1-3,15]. The demonstration of improvements in airway resistance in patients with mild asthma is important, because it suggests that even in the context of minimal evidence of lung function impairment using spirometry, there is significant reversible disease activity that can be detected by measures of lung function that are more sensitive than those routinely used in clinical practice.

Involvement of the small airways, even in patients with mild asthma is increasingly recognised [16]. Improved deposition of inhaled corticosteroid in the small airways may lead to a clinical benefit by reducing the persistent inflammation seen in these airways [16]. It has been suggested that combination therapy may result in enhanced delivery of inhaled corticosteroid to the peripheral airways [17]. Furthermore LABA's may have a direct bronchodilator effect in the small airways [17]. Frequency dependant changes in resistance and compliance have been demonstrated in small airway disease [18,19]. It has been suggested that oscillometry is a sensitive measure of small airway dysfunction as a range of frequencies are employed and changes in R5, X5 and RF may reflect small airway physiology [20-22]. Thus, the changes in IOS demonstrated in this study may indicate beneficial small airway effects of SFC. There was no improvement in the spirometric measurement of MMEF, which has also been suggested to be an indicator of small airway function. This is likely to be due to the increased variability of MMEF compared to IOS causing reduced sensitivity [1]. The use of IOS is increasing in clinical practice, and the current study shows a valuable application of this method for detecting subtle but important effects of drugs that cannot be measured by standard spirometry.

This study produced some additional findings concerning the use of combination therapy. We found that post dose pulmonary function measurements were stable for 4 weeks in both groups. There have been some concerns about desensitisation to the effects of long-acting beta_2 _agonists after prolonged treatment [23]. However, long-term studies up to 12 months duration have shown no evidence of tachyphylaxis or tolerance to the bronchodilator effects of salmeterol [24,25]. The current study also did not observe desensitisation, as 2 hr post dose lung function measurements in mild asthmatics treated with SFC were stable over a 4 week period.

There was a trend to an improvement in airway hyper reactivity (AHR) from baseline in the SFC treatment group but this was not significant. The current study did not enrol patients based on an inclusion criterion of AHR, and was not statistically powered to evaluate changes in AHR, as AHR was a secondary endpoint. Further studies powered to assess AHR as a primary endpoint are required to assess any potential benefit of SFC compared to FP alone on AHR in this patient population. However, a recent study has shown in mild to moderate patients that SFC improved AHR to a significantly greater extent than FP or salmeterol alone [26].

## Conclusion

It is recognised that a substantial number of patients have mild persistent asthma associated with significant morbidity [27,28]. We have clearly shown that SFC has beneficial effects on lung function in patients with mild asthma that are not observed with ICS alone. These benefits of SFC therapy were sensitively demonstrated by body plethysmography and IOS. Further long term studies in mild asthma are required to ascertain the relationship between the improvements in lung function observed in the current study using SFC and clinical parameters such as symptoms and exacerbation rates.

## Competing interests

Catherine Houghton has received travel grants from GlaxoSmithKline (GSK) and Astra Zeneca (AZ) and support for a conference from Chiesi. Naomi Lawson has no financial conflict of interest to declare. Zoe Borrill has received travel grants from GlaxoSmithKline (GSK) and support for a conference from Chiesi. Claire Wixon and Sally Yoxall are employees of GSK the manufacturer of SFC. Ashley Woodcock has received consultancy fees from GSK, Chiesi, Novartis, Schering Plough and Oriel Pharmaceuticals, research grants from GSK, Chiesi and Schering and support for conference attendance from GSK. Dave Singh has received lecture fees from AZ,GSK and Merck Sharp Dome, research grants fromGSK and AZ andsupport for conference attendance from Boehringer and AZ.

## Authors' contributions

All authors participated in the study design, CMH, NL and ZB coordinated the study and collected the data. SY performed the statistical analysis. CMH, CW, SY, DS and AAW analysed and interpreted the data. CMH drafted the manuscript and all authors read and approved the final manuscript.
